# Calling for a rapid recognition and response program for stroke in China

**Published:** 2016-09-20

**Authors:** Jing Zhao, Renyu Liu

**Affiliations:** 1 Department of Neurology, Minhang Hospital, Fudan University, Shanghai, China; 2 Department of Anesthesiology and Critical Care, Perelman School of Medicine at the University of Pennsylvania, Philadelphia, PA USA

**Keywords:** Stroke, FAST, China, Education, outcome, rapid recognition, prehospital delay

## Abstract

In this article, we provide evidences indicating that a a rapid recognition and response program based on FAST (face, Arm, Speech, Time) for stroke suitable for China is desperately needed.

China has the largest population in the world with over 1.38 billion people. There are more than 2.5 million new stroke patients each year with an increasing trend. The mean age for the first stroke is in the 66–70 years old range, younger than that in well-developed countries.[1–3] Based on the third Chinese National Survey on the Cause of Death, stroke is now the leading cause of death and disability, resulting in 117% annual increase in cost of care.[1–3] To reduce stroke incidence, and to minimize stroke related mortality and morbidity, both novel educational and therapeutics strategies are urgently needed.

Stroke can result in death and miserable life-long disabilities, significant burden to the patient, patient family, and the society. Death and disabilities could be potentially avoided if the treatment is initiated immediately after the onset of stroke, especially for the ischemic stroke. Intravenous thrombolytic therapy with recombinant tissue plasminogen activator (rtPA) has been a standard of care for ischemic stroke in most clinical settings. However, the therapy must be initiated within a very narrow therapeutic window (0–4.5 hours).[4] However, pre-hospital delay due to inadequate rapid stroke recognition and inadequate utilization of emergent medical service is very common across the world with a wide range of differences.[5–8] Prehospital delays are more prominent in developing countries where a robust stroke recognition and response system does not exist. Although intravenous thrombolytic therapy is available in China, studies indicated that only 1.6–4.0 % of ischemic stroke patients received such thrombolytic therapy due to significant prehospital delay with the median time of the delay has 15 hours. [9] A recent study found critical factors: 1) very few stroke victims (16.9%) recognized the initial signs of stroke; 2) less than 19% of victims used emergency medical service to transport them to hospitals in a timely manner; 3) ambulance physicians was able to diagnose 67% of stroke cases. [10] These data have at least two important implications in China: 1) education for stroke awareness and rapid recognition is urgently needed; 2) a mechanism to trigger emergent medical services immediately after the onset of the stroke is also needed.[9, 11]

To reduce prehospital delay, the “FAST” (“Face”, “Arm”, “Speech”, “Time”) was used as a mnemonic term based on the Cincinnati Pre-Hospital Stroke Scale published in 1999[12] to capture the most common signs of stroke in a timely manner.[13, 14] In 2009, the Department of Health in England launched a “FAST” mass media campaign, and it was associated with significant breakthroughs in stroke awareness, rapid recognition, and outcome.[15] Various stroke educational programs using “FAST” have now been developed across the world due to the effectiveness of “FAST” in reducing prehospital delay and increasing emergency medical service system utilization.

[16–18] In the recent SWIFT PRIME Multi-center Randomized Controlled Trial, the median time from the stroke symptom onset to arrival at the emergency department was less than 2 hours (109·5 min with interquartile range of 54–192.5 min).[19] Rapid stroke recognition with immediate triggering of medical emergency service played significant role.

Experts in China has recognized that the importance to use educational tools to increase stroke awareness. Organized by the Chinese Stroke Association, the “Red Bracelet Volunteer Group” was established in 2015 to promote stroke risk factor education and related medical volunteering services. More than 395 hospitals and 13400 volunteers across China have joined this group to date; more are predicted to join with expanded media coverage. However, a rapid recognition and response program for stroke is still missing. Some efforts have been made to implant “FAST” to be used in China as indicated in Figure 1 using various media tools. In one of the video media educational tool as indicated in the right panel of Figure 1, the “FAST” was used as a mnemonic tool without adaptation to the Chinese characters, only estimated about 5000 people viewed this educational video and material after its publication from Oct 10, 2015 in one year of period of time. The effectiveness is also unknown. Such unsatisfactory result is predictable because “FAST” is specifically designed for those who can understand English, and thus, direct implementation of “FAST” will be difficult in non-English speaking countries and regions, due to language barriers. Such barriers will be more prominent if there are deficiencies in awareness of the importance of using available emergency medical systems to rapidly transfer stroke victims to the hospital.

It is important to note that language and interpretation barriers exist for the “FAST” program even in English speaking countries. In the recent study evaluating the effectiveness of the national “FAST” campaign in Birmingham, England, surveys found that only 60.2% of the people were aware of the “FAST” campaign. Further, many people did not know what the acronym stood for.[20] Among the nearly 1·4 billion people in China, only about 0·8% (i.e., up to 10 million people excluding Hong Kong) of the population is considered capable of speaking English.[21] Thus, it is very clear that direct usage of “FAST” will be ineffective. A simple translation of the acronym “FAST” from English into Chinese does not work well either because the Chinese characters for *face, arm, speech* and *time* do not represent an interpretable word or memorable phrase. Thus, herein, we believe that a strategy based on the concept of “FAST” to improve stroke awareness and rapid identification, and to trigger emergency medical service utilization in a timely manner in China is urgently needed.

However, a creative strategy based on the concept of “FAST” is urgently needed. We welcome further discussion on this topic to identify effective educational tool for the public domain in China to help the increasing stroke victims in China for two critical issues: 1) rapid recognition of the stroke victims; 2) trigger the emergency medical service system in a timely manner to transport the victim to the hospital without delay.

## Figures and Tables

**Figure 1:**
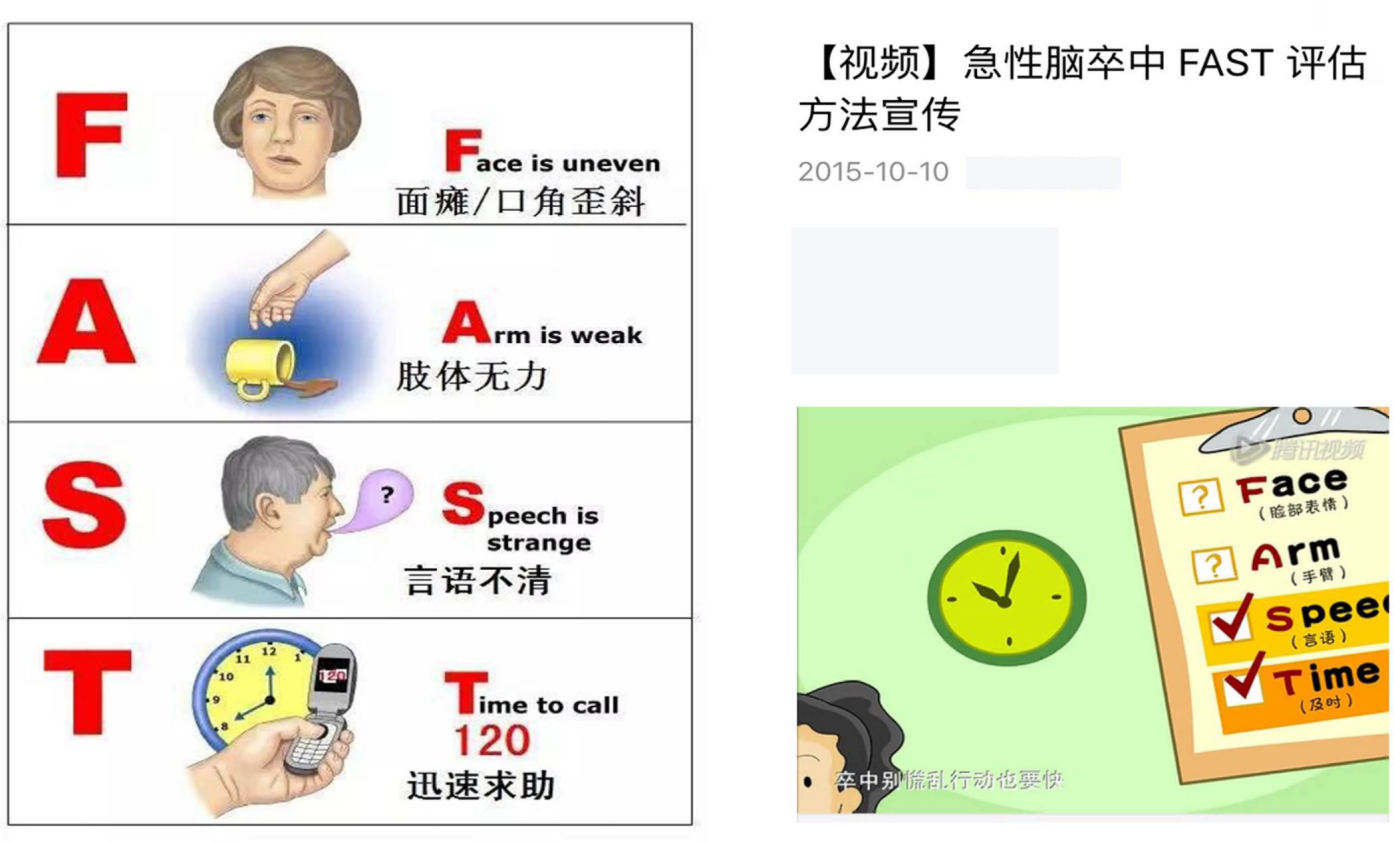
Current FAST education China Left Panel: An educational material using “FAST” with some simple Chinese explanation; right panel: An educational video using FAST with simple Chinese explanation. The title of the material is “Education using FAST evaluation for acute stroke”. The mnemonic English term “FAST” was left as it is. It is indicated that the material is published on Oct 10, 2016. All the above materials are available free online.
